# *MBE* Emerging Classics 2022

**DOI:** 10.1093/molbev/msab357

**Published:** 2022-01-28

**Authors:** Heather Rowe

**Affiliations:** Institute for Genomics and Evolutionary Medicine, Temple University, Philadelphia, PA, USA

Since 2013, *Molecular Biology and Evolution* has celebrated papers with powerful impact on our community as reflected by accumulated citations. Below, we highlight ten discoveries, five methods, and five resource publications as “Emerging Classics” based on citations accrued per fractional years since publication. Articles within categories are listed in alphabetical order based on the family name of the first author. Total citation counts were obtained from Web of Science on November 30, 2021. We congratulate these authors on the significance of their contributions and look forward to publishing many high-impact articles in the years to come.

## Discoveries

*Large variation in the ratio of mitochondrial to nuclear mutation rate across animals: implications for genetic diversity and the use of mitochondrial DNA as a molecular marker* (2017) Allio and colleagues in Volume 34(11) Pp. 2762–2772.

*Evolution of DNA methylation across insects* (2017) Bewick and colleagues in Volume 34(3) Pp. 654–665.

*The rice paradox: multiple origins but single domestication in Asian rice* (2017) Choi and colleagues in Volume 34(4) Pp. 969–979.

*Deciphering the routes of invasion of Drosophila suzukii by means of ABC random forest* (2017) Fraimout and colleagues in Volume 34(4) Pp. 980–996.

*Between a pod and a hard test: the deep evolution of amoebae* (2017) Kang and colleagues in Volume 34(9) Pp. 2258–2270.

*Genomic analysis of European Drosophila melanogaster populations reveals longitudinal structure, continent-wide selection, and previously unknown DNA viruses* (2020) Kapun and colleagues in Volume 37(9) Pp. 2661–2678.

*Adaptation of S. cerevisiae to fermented food environments reveals remarkable genome plasticity and the footprints of domestication* (2018) Legras and colleagues in Volume 35(7) Pp. 1712–1727.

*Genomic evidence for complex domestication history of the cultivated tomato in Latin America* (2020) Razifard and colleagues in Volume 37(4) Pp. 1118–1132.

*Extreme genomic CpG deficiency in SARS-CoV-2 and evasion of host antiviral defense* (2020) Xia in Volume 37(9) Pp. 2699–2705.

*Evolution of Rosaceae fruit types based on nuclear phylogeny in the context of geological times and genome duplication* (2017) Xiang and colleagues in Volume 34(2) Pp. 262–281.

## Methods

*Genomic infectious disease epidemiology in partially sampled and ongoing outbreaks* (2017) Didelot and colleagues in Volume 34(4) Pp. 997–1007.

*The unreasonable effectiveness of convolutional neural networks in population genetic inference* (2019) Flagel and colleagues in Volume 36(2) Pp. 220–238.

*A fast likelihood method to reconstruct and visualize ancestral scenarios* (2019) Ishikawa and colleagues in Volume 36(9) Pp. 2069–2085.

*New methods to calculate concordance factors for phylogenomic datasets* (2020) Minh and colleagues in Volume 37(9) Pp. 2727–2733.

*Theoretical foundation of the RelTime method for estimating divergence times from variable evolutionary rates* (2018) Tamura and colleagues in Volume 35(7) Pp. 1770–1782.

## Resources

*UFBoot2: improving the ultrafast bootstrap approximation* (2018) Hoang and colleagues in Volume 35(2) Pp. 518–522.

*MEGA X: molecular evolutionary genetics analysis across computing platforms* (2018) Kumar and colleagues in Volume 35(6) Pp. 1547–1549.

*PartitionFinder 2: new methods for selecting partitioned models of evolution for molecular and morphological phylogenetic analyses* (2017) Lanfear and colleagues in Volume 34(3) Pp. 772–773.

*IQ-TREE 2: new models and efficient methods for phylogenetic inference in the genomic era* (2020) Minh and colleagues in Volume 37(5) Pp. 1530–1534.

*DnaSP 6: DNA sequence polymorphism analysis of large data sets* (2017) Rozas and colleagues in Volume 34(12) Pp. 3299–3302.

